# Synergistic Effect between Eugenol and 1,8-Cineole on Anesthesia in Guppy Fish (*Poecilia reticulata*)

**DOI:** 10.3390/vetsci11040165

**Published:** 2024-04-06

**Authors:** Saransiri Nuanmanee, Preeyanan Sriwanayos, Khemmapat Boonyo, Wasana Chaisri, Banthita Saengsitthisak, Preechaya Tajai, Surachai Pikulkaew

**Affiliations:** 1 Songkhla Aquatic Animal Health Research and Development Center, Department of Fisheries, Songkhla 90100, Thailand; 2 Aquatic Animal Health Research and Development Division, Department of Fisheries, Bangkok 10900, Thailand; 3 Bureau of Disease Control and Veterinary Services, Department of Livestock Development, Bangkok 10400, Thailand; 4 Faculty of Veterinary Medicine, Chiang Mai University, Chiang Mai 50100, Thailand; 5 Faculty of Pharmacy, Payap University, Chiang Mai 50000, Thailand; 6 Department of Forensic Medicine, Faculty of Medicine, Chiang Mai University, Chiang Mai 50200, Thailand; 7 Research Center of Producing and Development of Products and Innovations for Animal Health and Production, Chiang Mai University, Chiang Mai 50100, Thailand

**Keywords:** eugenol, 1,8-cineole, anesthesia, synergistic effect, guppy

## Abstract

**Simple Summary:**

This study examined the synergistic effect of eugenol and 1,8-cineole (eucalyptol) on anesthesia in female guppy fish. The results showed that eugenol induced fish anesthesia at concentrations of 50 and 70 mg/L, with durations of 256.5 and 171.5 s, respectively. 1,8-cineole did not induce fish anesthesia. Combining eugenol with 1,8-cineole resulted in the faster induction of anesthesia and a longer recovery time. This study concluded that eugenol and 1,8-cineole work better together as anesthetics, demonstrating the safety of using these agents on guppy fish.

**Abstract:**

This study aimed to evaluate the synergistic effect between eugenol and 1,8-cineole on anesthesia in female guppy fish (*Poecilia reticulata*). Experiment I evaluated the concentrations of 0, 12.5, 25, 50, and 75 mg/L of eugenol and 0, 100, 200, 300, and 400 mg/L of 1,8-cineole for times of induction and recovery from anesthesia. Experiment II divided fish into 16 study groups, combining eugenol and 1,8-cineole in pairs at varying concentrations, based on the dosage of the chemicals in experiment I. The results of the anesthesia showed that eugenol induced fish anesthesia at concentrations of 50 and 70 mg/L, with durations of 256.5 and 171.5 s, respectively. In contrast, 1,8-cineole did not induce fish anesthesia. In combination, using eugenol at 12.5 mg/L along with 1,8-cineole at 400 mg/L resulted in fish anesthesia at a time of 224.5 s. Increasing the eugenol concentration to 25 mg/L, combined with 1,8-cineole at 300 and 400 mg/L, induced fish anesthesia at times of 259.0 and 230.5 s, respectively. For treatments with eugenol at 50 mg/L combined with 1,8-cineole at 100 to 400 mg/L, fish exhibited anesthesia at times of 189.5, 181.5, 166.0, and 157.5 s. In the case of eugenol at 75 mg/L, fish showed anesthesia at times of 175.5, 156.5, 140.5, and 121.5 s, respectively. The testing results revealed that 1,8-cineole as a single treatment could not induce fish anesthesia. However, when supplementing 1,8-cineole in formulations containing eugenol, fish exhibited a significantly faster induction of anesthesia (*p* < 0.05). Furthermore, all fish that underwent anesthesia were able to fully recover without any mortality. However, the shorter anesthesia duration resulted in a significantly prolonged recovery time. In conclusion, eugenol and 1,8-cineole work better together as anesthetics than when used separately, and demonstrated the safety of using these anesthetic agents on guppy fish.

## 1. Introduction

Guppy fish, scientifically known as *Poecilia reticulata*, is a colorful and viviparous species of freshwater fish that is gaining popularity among enthusiasts of aquarium keeping. In addition, they hold the second position in Thailand’s ornamental fish export market, following bettas. According to the Department of Fisheries Thailand, Thailand’s export of guppy fish reached 9.6 million individuals, with a total value of 32.5 million baht in 2022. Fish are susceptible to stress when they are handled and transported, which can result in injury or mortality. However, susceptibility to stress varies among fish species, influencing their rates of mortality and injury [1]. Using anesthesia in transportation can increase the carrying capacity, which is the margin of the environment’s ability to provide the necessary resources to sustain fish life, by reducing fish metabolism, oxygen consumption, and nitrogenous waste excretion. Moreover, anesthesia is always used for immobilizing aquatic animals, resulting in less stressful handling.

Traditional chemical fish anesthetics, such as urethane, ether, and chloroform, are now restricted due to their carcinogenic properties, whereas some natural compounds are safe and can be used as food additives, while also possessing sedative properties in aquatic animals, such as clove oil, which contains the active ingredient eugenol [2]. Eugenol, an aromatic oil extracted from clove (*Syzygium aromaticum*), exhibits potential applications in aquatic animals and has been extensively used in many research projects [3,4]. Several plants, including *Ocimum basilicum*, *O. canum*, and *O. sanctum*, have been extracted for their essential oils to study their anesthetic properties in fish [5]. Recently, 1,8-cineole (eucalyptol), a monoterpene cyclic ether abundantly found in nature, has been reported to have anesthetic effects in many fish species, such as common carp [6], koi carp [7], and Nile tilapia [8].

In a combination regimen, pharmacodynamic drug interactions occur when the effect of one medication influences the pharmacological impact of another [9]. Subsequently, the fish were exposed to two or more chemicals simultaneously, resulting in observed health effects and interactions. Pharmacodynamics describes numerous processes through which interactions might occur and is often categorized as synergistic, additive, or antagonistic [10]. A synergistic effect of a mixture of anesthetics on fish species was investigated in grass puffer (*Takifuku niphobles*), using clove oil and lidocaine hydrochloride. The result revealed a shorter time to anesthesia and a more rapid recovery of stress responses compared to each anesthetic alone [11]. Therefore, the objectives of this study were to assess the anesthetic response, determine the suitable concentration ranges of eugenol and 1,8-cineole, and examine the potential synergistic effect of a combination of these two anesthetics on guppy fish.

## 2. Materials and Methods

### 2.1. Chemicals and Preparation of Chemicals

Absolute ethanol was from RCI Labscan™ (Pathumwan, Bangkok, Thailand). The pure compound of eugenol (≥98%) was from Sigma-Aldrich (St. Louis, MO, USA), and 1,8-cineole (≥98%) was from Merck Millipore (Billerica, MA, USA). For the animal study, eugenol and 1,8-cineole were freshly prepared by diluting with 100% ethanol at a 1:9 (*v*/*v*) ratio and used within 2 h of preparation.

### 2.2. Animals and Rearing Conditions

One hundred and forty-four healthy adult female guppies, with an average weight of 1.02 ± 0.14 g and a total length of 4.74 ± 0.21 cm, were obtained from an ornamental fish store located in Chiang Mai, Thailand. The fish were quarantined with contagious diseases for two weeks in a 300-L glass tank. The dissolved oxygen (DO) levels in the tanks were maintained by means of constant aeration. During the quarantine period, dechlorinated tap water was monitored and changed daily (50%). Temperature and dissolved oxygen were measured using a DO meter (Model Y550A, YSI Incorporated, Yellow Springs, OH, USA) and a pH meter (CyberScan 500, Eutech Instruments Pte Ltd., Ayer Rajah Crescent, Singapore), and total ammonia and nitrite levels were determined using Tetra Test^®^ kits (Tetra Werke, Melle, Germany). The fish were fed daily at 9.00 a.m. and 4.00 p.m., until apparent satiation, with a commercial pelletized diet containing 47.5% crude protein (Tetra Werke, Melle, Germany) under natural light conditions. After the quarantine period, the fish were subjected to a fasting period of twelve hours before being transferred to the experimental glass tank.

### 2.3. In Vivo Anesthetic Activity

The fish were randomly divided into 2 experiments, including experiment I and experiment II; each study group contained six individual fish that were used only once (*n* = 6). Experiments were conducted in glass aquaria (10 × 10 × 15 cm) containing 1 L of dechlorinated water prepared for anesthesia or induction tanks.

For experiment I (10 study groups), the fish were moved to an induction tank that had diluted eugenol solutions with final concentrations of 0, 12.5, 25, 50, and 75 mg/L of eugenol and 0, 100, 200, 300, and 400 mg/L of diluted 1,8-cineole solutions. The concentrations of eugenol and cineole were selected based on the studies by Cunha et al., 2015, and Hoseini et al., 2020, respectively. The effect of the chemicals on fish anesthetics was investigated by determining the induction time to anesthesia, adapted from Coyle et al., 2004 [1], McFarland, 1959 [12], and Zahl et al., 2012 [13], which was observed after the fish reached the surgical stage or stage 3 (to determine this step, the caudal peduncle area of a breathing but non-swimming fish is gently crushed with a forceps; if the fish does not show any signs of involuntary muscular reactions or flap its fins, it has no pain reflex). After the anesthesia induction procedure was complete, the fish were transferred to the recovery tank, which contained 1 L of oxygenated water without anesthetic agents. The stage of recovery behavior was adapted from Iwama and Ackerman, 1994 [14] (see Table 1). A tank containing 0.36% (*v*/*v*) absolute ethanol (the same as the maximum amount used in the anesthetic tank) in dechlorinated water was used as a vehicle control for both experiments. For experiment II, the fish were divided into 16 study groups, combining eugenol and 1,8-cineole in pairs at varying concentrations, based on the dosage of the chemicals in experiment I. For both experiments, if the anesthetic induction time exceeded 600 s, the experimental animal was not considered to have entered anesthesia.

### 2.4. Data Analyses and Manuscript Language Editing

The normality assumption of the data was assessed using the Shapiro–Wilk test, while non-parametric data were presented in the median ± interquartile range, and the differentiation of induction time and recovery time was analyzed using the Kruskal–Wallis test and pairwise Mood’s median test. Statistical significance was determined at *p* < 0.05. GraphPad Prism version 8.3.0 for Windows (GraphPad Software, San Diego, CA, USA, www.graphpad.com, accessed on 28 March 2024) was used to determine the LogEC50, which represents the concentration of a substance required to produce a 50% response after a specified exposure time. The analysis employed the best-fit model, with R² serving as the indicator of goodness of fit. Plagiarism and grammar checks were performed using QuillBot version 15.1.1 (QuillBot, a Learneo, Inc. business, Chicago, IL, USA)

## 3. Results

### 3.1. Experiment I (Single Chemical)

#### 3.1.1. Induction Time of Each Chemical

The results from the use of eugenol at concentrations of 12.5, 25, 50, and 75 mg/L revealed that eugenol at a concentration of 12.5 mg/L could not induce fish to achieve stage 1 anesthesia. Meanwhile, at 25 mg/L, eugenol induced fish to reach stage 2 anesthesia, with induction times of 140 and 206.5 s for stages 1 and 2, respectively. At the higher concentrations of 50–75 mg/L, eugenol-induced fish entered stage 3 anesthesia, with the shortest induction time observed at 75 mg/L (39.00, 57.00, and 171.5 s for stages 1, 2, and 3, respectively) (Table 2).

In contrast, the use of 1,8-cineole at concentrations of 100, 200, 300, and 400 mg/L showed that fish exposed to 100 and 200 mg/L could not achieve stage 1 anesthesia. However, at 300 and 400 mg/L, 1,8-cineole induced fish to reach stage 2 anesthesia, and at 400 mg/L, fish exhibited the shortest induction time of 48.5 and 91.5 s for stages 1 and 2, respectively (Table 3). An ethanol concentration of 0.3% *v*/*v* did not induce sedation in the fish nor resulted in any adverse effects. 

#### 3.1.2. Recovery Time

Fish exposed to eugenol at a concentration of 50 mg/L exhibited a recovery time 1.51 times longer than that of the group treated with 75 mg/L of eugenol. Importantly, eugenol at concentrations of 12.5 and 25 mg/L, as well as 1,8-cineole at all concentration levels, failed to induce fish to achieve stage 3 anesthesia. Consequently, the assessment of recovery from anesthesia could not be conducted under these conditions (Table 2 and Table 3). There was no mortality during the experimental period.

### 3.2. Experiment II (Combination of Chemicals)

#### 3.2.1. Induction Time

It was observed that an anesthetic treatment containing eugenol can induce a higher level of anesthesia in fish with shorter induction times, as the concentration of 1,8-cineole was increased in the anesthetic combination. This is evident in the behavior of fish exposed to the combined anesthetic with eugenol at various concentration levels. For eugenol at a concentration of 12.5 mg/L, when combined with 100 mg/L of 1,8-cineole, fish were induced to achieve stage 1 anesthesia with an induction time of 132.5 s. When combined with 200 and 300 mg/L of 1,8-cineole, fish were induced to achieve stage 2 anesthesia with induction times of 166.0 and 109.0 s, respectively. When combined with 400 mg/L of 1,8-cineole, fish were induced to reach stage 3 anesthesia with an induction time of 224.5 s (Table 4). 

Similarly, for eugenol at a concentration of 25 mg/L, when combined with 1,8-cineole at 100 and 200 mg/L, fish were induced to achieve stage 2 anesthesia with induction times of 190.5 and 130.5 s, respectively. When combined with 300 and 400 mg/L of 1,8-cineole, fish were induced to reach stage 3 anesthesia with induction times of 259.0 and 230.5 s, respectively (Table 4). 

In determining the EC50, we found that, when used individually, 1,8-cineole had a value (log EC50) of 16.45. However, when combined with eugenol at 12.5 mg/L and 25 mg/L, the values were shifted to 1.39 and 2.44 at stage 1 of the induction time (Supplementary Figure S1). When used individually, 1,8-cineole had a value of 16.88 for stage 2 of the induction time, but when mixed with eugenol at 12.5 mg/L and 25 mg/L, it had values of 2.92 and 4.19, respectively (Supplementary Figure S2).

Furthermore, for eugenol at concentrations of 50 and 70 mg/L, when combined with 1,8-cineole at 100, 200, 300, and 400 mg/L, fish were induced to achieve stage 3 anesthesia at all concentrations, with induction times exhibiting significant decreasing trends with increasing 1,8-cineole concentrations (Table 4).

#### 3.2.2. Recovery Time

It was observed that an anesthetic formulation containing eugenol with an increased concentration of 1,8-cineole leads to longer recovery times. Specifically, for an anesthetic with eugenol at a concentration of 12.5 mg/L combined with 400 mg/L of 1,8-cineole, fish exhibited a recovery time of 213.5 s. For eugenol at a concentration of 25 mg/L combined with 300 and 400 mg/L of 1,8-cineole, no significant differences in recovery times were observed. Additionally, for eugenol at concentrations of 50 and 70 mg/L, when combined with 1,8-cineole at 100, 200, 300, and 400 mg/L, fish exhibited longer recovery times, correlating with the increasing concentration of 1,8-cineole (Table 4). During the experimental periods, there was no mortality.

## 4. Discussion

Several studies have investigated the active ingredients in plant compounds that induce anesthesia in various fish species, with eugenol being a widely used substance. Eugenol serves the purpose of providing light sedation for transportation and achieving deep anesthesia for surgical procedures. Furthermore, it is noted for its cost-effectiveness [1]. The findings of this study indicated that eugenol concentrations of 12.5 and 25 mg/L failed to induce anesthesia in female guppy fish, consistent with previous studies on guppy fish reporting induction at concentrations starting at 50 mg/L [15]. In this study, we observed that eugenol at a concentration of 75 mg/L induced anesthesia faster than at 50 mg/L, which is consistent with previous findings in female guppies [15]. It is possible that the time of drug absorption may affect the amount of drug in other tissues and may later interact with the central nervous system. This issue may require a detailed study on the distribution phase and elimination phase in fish exposed to eugenol at different time intervals. Additionally, increasing the eugenol concentration to 100, 125, and 150 mg/L resulted in fish requiring less time for induction, as reported by Canha et al., 2015 [4]. This observation corresponds with findings in Guaru fish (*Poecilia vivipara*), where eugenol concentrations of 100 and 200 mg/L gradually decreased the induction time [16]. Therefore, it is hypothesized that eugenol exhibits a dose-dependent effect on induction time.

Regarding recovery time, eugenol at a concentration of 75 mg/L demonstrated a faster recovery time compared to 50 mg/L, which is like another study conducted on female guppy fish [4]. However, these results differ from the study of Santos et al. in 2017, where the recovery time with 50 mg/L eugenol ranged from 64.76 to 176.23 s, and with 75 mg/L, it fell within the range of 64.77 to 192.98 s. This inconsistency may be attributed to the fact that the experiment was conducted on four different sizes of guppy fish, weighing 0.06, 0.21, 0.36, and 0.63 g, resulting in significant differences in induction time and recovery time among the groups [15]. This experiment used female guppy fish as the model for the study to investigate the drug’s effects and minimize confounding factors from the animals. However, the concentration required to sedate both male and female fish differed. According to the study by Cunha et al., 2015, females and males were sedated with eugenol at concentrations of 75 and 125 mg/L, respectively [4]. Additionally, a study on doctor fish (*Garra rufa*) suggested that eugenol at concentrations of 25 and 50 mg/L is suitable for general aquaculture procedures, while 75 mg/L induces sedation and proves suitable for specific tasks requiring stability, such as blood collection [17]. Therefore, the study emphasizes the importance of controlling factors such as sex, size, and species when investigating anesthesia and recovery processes.

1,8-cineole has been studied for its anesthetic properties in various fish species, with a higher concentration of 400 mg/L generally considered effective for achieving suitable induction times. It has been observed that the administration of 1,8-cineole at 400 mg/L results in common carp (*Cyprinus carpio*) and caspian trout (*Salmo caspius*) experiencing induction times of 460 and 180 s, respectively [18,19]. A review of studies on monoterpines for fish anesthesia, on the other hand, found that the best concentration of 1,8-cineole to induce anesthesia in adult rainbow trout (*Oncorhynchus mykiss*) and caspian trout (*Salmo caspius*) was around 461.0 and 522.6 mg/L, with induction times of 162 and 126 s [20]. Contrastingly, the results from this study indicated that a concentration of 1,8-cineole at 400 mg/L could not induce anesthesia in guppy fish within 600 s. For those intending to investigate the anesthesia-inducing concentration of 1,8-cineole in guppy fish, it is recommended to study concentrations higher than 400 mg/L. However, caution should be exercised not to exceed the LC50 level, as 1,8-cineole demonstrates toxicity towards guppy fish, indicating LC50 values of 3997.07 and 1701.93 mg/L for male and female fish, respectively [21].

Additionally, eugenol and lidocaine have been used in river puffer (*Takifugu obscurus*) and tiger puffer (*T. rubripes*). The study revealed that increasing the level of lidocaine significantly reduced the induction time [22]. Currently, there are no existing studies on the combination of eugenol and 1,8 cineole formulations in fish. In the present study, the efficacy of eugenol and 1,8-cineole was investigated, revealing that the addition of 1,8-cineole to the eugenol formulation led to deeper anesthesia. For instance, in the eugenol 12.5 mg/L formulation, fish showed no response to anesthesia. However, with the addition of 1,8-cineole at concentrations of 100, 200, and 400 mg/L, fish were induced to achieve stages 1, 2, and 3 of anesthesia, respectively. 

Several studies have employed plant extracts to investigate anesthetic applications in koi carp (*Cyprinus carpio*). These experiments revealed that koi carp can reach stage 3 anesthesia upon exposure to essential oils from *Ocimum* sp. at a concentration of 100 mg/L and *Alpinia galanga* at a concentration of 200 mg/L, which contain eugenol, 1,8-cineole, and various other compounds [5,7]. Research on plant extracts holds the potential to advance the development of effective anesthetic formulations for aquatic animals. However, it is crucial to analyze the quantity of active ingredients each time, even within the same genus, as different species may have varying compositions. For example, within the Melaleuca genus, including *Melaleuca linariifola*, *M. bracteata*, and *M. leucadendron*, 1,8-cineole proportions are 61.1%, 0.2%, and 0.1%, respectively, while their eugenol proportions are 0.3%, 0.5%, and 0.1%, respectively [23]. Furthermore, even within the same plant species, the geographic location of cultivation can lead to variations in essential oil composition. For instance, a study on essential oil from *Tasmanian bluegum* (*Eucalyptus globulus*) cultivated in various countries reported 1,8-cineole compositions ranging from 43.18% to 85.82% [24].

Although their individual components are thoroughly studied, the mechanisms by which medications interact in clinical combination regimens still need to be better understood. Two possible ways in which medications in a combination therapy could interact are (a) if one drug just enhances the effects of the other, or (b) if the two drugs work together to produce effects that are not present in either drug individually. General anesthesia reduces activity in the central nervous system (CNS), leading to unconsciousness and a complete loss of sensation. Following this, various levels of pain relief and muscle relaxation may occur. The anesthetic agent added to water is absorbed through the gills and quickly spreads to the secondary lamellae, arterial blood, and ultimately the brain [25]. While the single-drug components of these medications have been extensively researched, the processes via which they interact in clinical combination regimens remain inadequately understood. Generally, anesthetics act as gamma-aminobutyric acid (GABA) type A receptor (GABA_A_) agonists, N-methyl-D-aspartate receptor antagonists, α2-Adrenoceptor agonists, or dopaminergic receptor antagonists [26]. The mechanism of eugenol’s action is believed to involve the GABA_A_, which inhibits nociception (producing an analgesic or anesthetic effect) and results in the specific blockade of nicotinic receptors (leading to a paralytic effect) [27]. In fish, inhibitory neurotransmitters in the central nervous system, such as GABA, the GABA-synthesizing enzyme glutamic acid decarboxylase 65 (GAD65), and the receptor subunits GABA_α1_ and GABA_B1_, are present, as in mammals [28]. Consequently, these interactions result in sedation or unconsciousness. While 1,8-cineole directly affects the Na+ channels of neurons in the superior cervical ganglion in mammals, potentially acting as a primary factor in excitability blockade, its mechanism of action seems to be the classic local anesthetics rather than binding to GABA_A_ receptors [26,29]. While the inability of 1,8-cineole to induce anesthesia in fish occurred during stage 3, the LogEC50 values observed in stages 1 and 2 indicated a reduction in anesthetic time when eugenol and eucalyptol were administered. The mechanisms of action of both substances exhibited contrasts. As a result, it is possible to conclude that eugenol and 1,8-cineole have a synergistic effect when administered together. 

Indeed, ethanol reduces the need for anesthesia and can potentially interact with one or both substances in a way that is either additive or synergistic. In this investigation, the fish exposed to the ethanol concentration showed no signs of sedation or any adverse effects. The presence of synergy, if it exists, cannot be definitively associated with unexplained ethanol effects at significantly higher concentrations.

In conclusion, our findings demonstrate the addition of 1,8-cineole to formulations containing eugenol resulted in a notable acceleration of anesthetic induction in guppy. The ideal anesthetics should possess characteristics that induce fish to stage 1, 2, and 3 anesthesia within time intervals of 30–45 s, 45–90 s, and 120–240 s, respectively. This is crucial because an excessively rapid induction may indicate an overdose, leading to potential toxicity, while prolonged induction times may suggest an insufficient dose [30]. Additionally, an optimal recovery time should not exceed 300 s [31]. The findings on induction and recovery times indicated that single-drug formulations do not possess the ideal anesthetic properties for guppy fish. In contrast, formulations combining eugenol and 1,8-cineole in ratios of 25:400, 50:100, and 75:100 demonstrate ideal anesthetic characteristics. We anticipate that this study will be beneficial for exploring the combined use of these two substances. However, further research into pharmacokinetics, pharmacodynamics, and the toxicity of this combination drug in diverse animal models and various environmental conditions is needed. This will contribute to the development of an effective anesthetic formulation in the future.

## Figures and Tables

**Table 1:** The anesthetic and recovery stages of guppy fish.

Stages		Description	Details
Normal		Normal	Normal swimming
Anesthetic	1	Sedation	Reduced swimming activity
	2	Excitatory stage	Reduced swimming activity and showing partial loss of equilibrium
	3	Surgical stage	Stopped swimming activity, experiencing a total loss of equilibrium and pain reflex
	4	Death stage	Medullary collapse and stopped respiration
Recovery	1	Starting movement	Starting movement of fins
	2	Regular breathing	Partial loss of equilibrium with normal breathing
	3	Total recovery	Normal swimming

**Table 2:** The median (M) and interquartile range (IQR) of the induction and recovery time of eugenol in female guppy (*n* = 6).

Eugenol (mg/L)	Induction Time (s)						Recovery Time (s)	
	Stage 1		Stage 2		Stage 3			
	M	IQR	M	IQR	M	IQR	M	IQR
12.5								
25	140.0 ^a^	113.25–171.25	206.5 ^a^	200.75–207.00				
50	84.0 ^b^	70.75–90.50	101.0 ^b^	98.00–107.75	256.5 ^a^	250.00–258.50	420.0 ^a^	408.00–432.75
75	39.0 ^c^	38.00–43.00	57.0 ^c^	57.00–63.00	171.5 ^b^	159.00–177.25	278.0 ^b^	250.50–282.25

Note: Different superscript letters in a column (^a^, ^b^, and ^c^) indicate a difference (*p* < 0.05) for a pairwise comparison between the predicted values for each group.

**Table 3:** The median (M) and interquartile range (IQR) of the induction and recovery time of 1,8-cineole in female guppy (*n* = 6).

1,8-Cineole (mg/L)	Induction Time (s)						Recovery Time (s)	
	Stage 1		Stage 2		Stage 3			
	M	IQR	M	M	IQR	IQR	M	IQR
100								
200								
300	105.0 ^a^	93.50–106.75	139.0 ^a^	128.50–160.00				
400	48.5 ^b^	44.75–54.50	91.5 ^b^	79.75–101.00				

Note: Different superscript letters in a column (^a^ and ^b^) indicate a difference (*p* < 0.05) for a pairwise comparison between the predicted values for each group.

**Table 4:** The median (M) and interquartile range (IQR) of the induction and recovery time of combination anesthetics in female guppy (*n* = 6).

Concentration (mg/L)		Induction (s)						Recovery Time (s)	
Eugenol	1,8-Cineole	Stage 1		Stage 2		Stage 3			
		M	IQR	M	IQR	M	IQR	M	IQR
12.5	100	132.5 ^a^	114.00–148.75						
	200	83.0 ^bc^	70.50–93.25	166.0 ^a^	154.50–179.00				
	300	61.5 ^bd^	55.25–70.75	109.0 ^b^	106.00–120.25				
	400	52.5 ^bd^	42.50–66.25	84.0 ^c^	76.25–88.75	224.5 ^b^	214.00–240.25	213.5 ^b^	202.50–218.50
25	100	79.5 ^c^	76.00–88.25	190.5 ^d^	187.25–206.50				
	200	77.5 ^bc^	64.50–80.00	130.5 ^b^	121.25–136.00				
	300	50.0 ^d^	49.25–57.50	92.5 ^c^	81.75–101.00	259.0 ^a^	255.75–267.50	191.0 ^ab^	181.00–198.75
	400	31.5 ^e^	30.25–33.50	70.0 ^c^	66.00–81.50	230.5 ^b^	217.00–238.75	189.0 ^ab^	178.50–207.00
50	100	31.5 ^e^	30.00–36.75	71.0 ^ce^	60.50–84.50	189.5 ^c^	186.00–190.00	201.0 ^ab^	186.25–212.75
	200	27.5 ^ef^	25.50–31.75	51.5 ^ef^	45.75–58.75	181.5 ^c^	178.75–194.00	236.0 ^b^	222.75–244.75
	300	28.0 ^ef^	27.00–30.50	42.0 ^fg^	37.50–47.25	166.0 ^d^	159.00–169.25	241.0 ^c^	240.25–248.50
	400	26.0 ^fg^	23.00–27.50	39.0 ^fgh^	35.00–43.75	157.5 ^de^	144.25–163.25	245.5 ^cd^	240.25–280.75
75	100	37.0 ^bdef^	27.25–61.00	45.5 ^cefgh^	37.50–71.50	175.5 ^de^	148.75–179.00	192.5 ^ab^	186.25–217.50
	200	30.0 ^ef^	25.50–36.75	35.5 ^h^	31.25–36.75	156.5 ^de^	147.50–159.50	249.5 ^cd^	232.50–269.50
	300	27.0 ^ef^	25.50–29.25	32.5 ^gh^	30.50–36.75	140.0 ^e^	135.50–143.75	263.5 ^cd^	235.75–277.00
	400	18.5 ^g^	18.00–20.50	32.5 ^h^	30.00–35.00	121.5 ^f^	120.25–123.50	266.5 ^cd^	249.00–293.00

Note: Different superscript letters in a column (^a^ to ^h^) indicate a difference (*p* < 0.05) for a pairwise comparison between the predicted values for each group.

## Data Availability

The data presented in this study are available upon request from the corresponding author. The data are not publicly available due to the privacy policy of the authors’ institution.

## Supplementary Materials

The following supporting information can be downloaded at: https://www.mdpi.com/article/10.3390/vetsci11040165/s1, Figure S1. The figure represents LogEC50, which represents the concentration of a substance required to produce a 50% response after a specified exposure time (at 12.5 mg/L and 25 mg/L of eugenol) in stage 1 of induction; Figure S2. The figure represents LogEC50, which represents the concentration of a substance required to produce a 50% response after a specified exposure time (at 12.5 mg/L and 25 mg/L of eugenol) in stage 2 of induction.
