# Supplementary material for: Synergistic Effect between Eugenol and 1,8-Cineole on Anesthesia in Guppy Fish (Poecilia reticulata)
Source: Vet Sci. 2024 Apr 6;11(4):165. doi: 10.3390/vetsci11040165 (PMC11054333; doi:10.3390/vetsci11040165)
Supplement: Supplementary file 1 [file vetsci-11-00165-s001.zip › vetsci-2900002-supplementary.pdf]

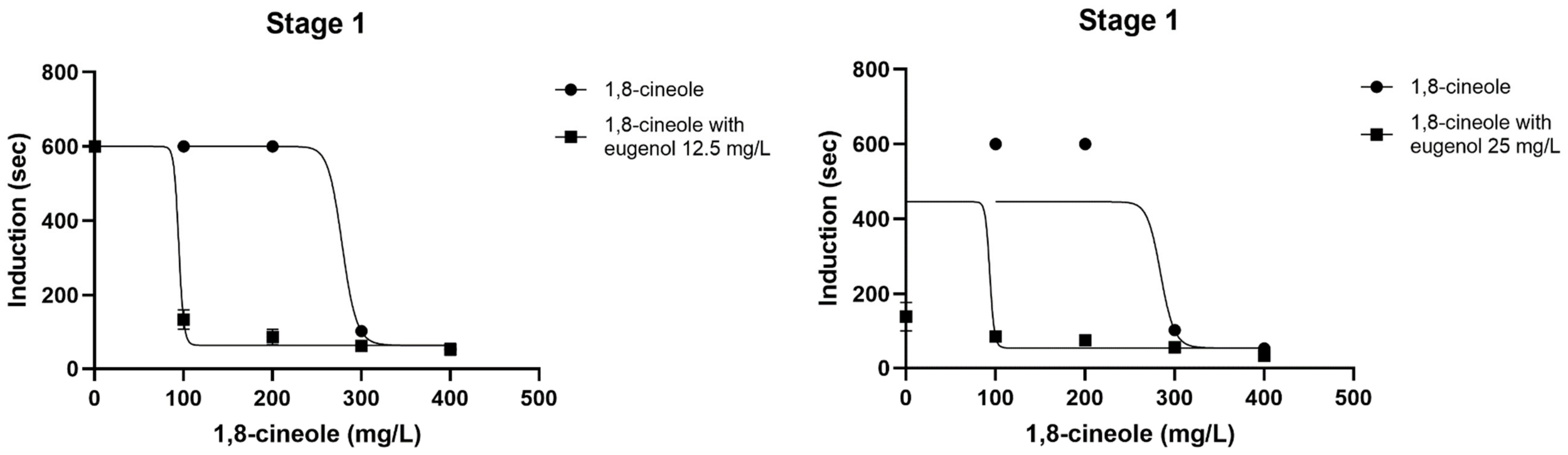

Supplementary Figure S1. The figure represents LogEC50, which represents the concentration of a substance required to produce a 50% response after a specified exposure time (at 12.5 mg/L and 25 mg/L of eugenol) in stage 1 of induction.

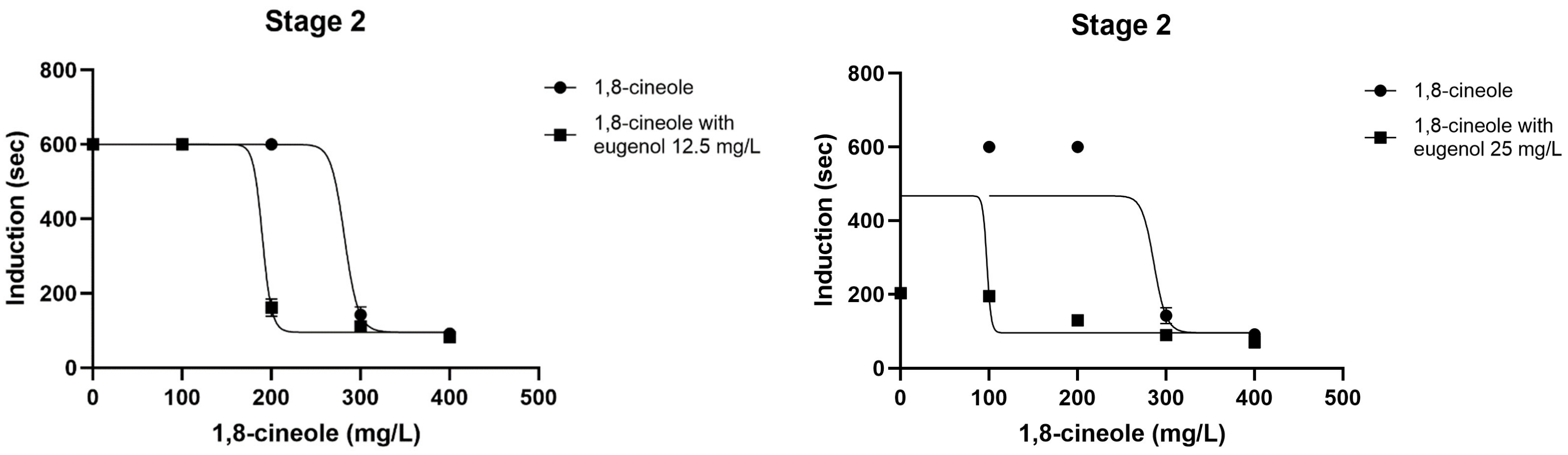

Supplementary Figure S2. The figure represents LogEC50, which represents the concentration of a substance required to produce a 50% response after a specified exposure time (at 12.5 mg/L and 25 mg/L of eugenol) in stage 2 of induction.
